# Maternal Mental Health and Its Association with Infant Growth at 6 Months in Ethnic Groups: Results from the Born-in-Bradford Birth Cohort Study

**DOI:** 10.1371/journal.pone.0030707

**Published:** 2012-02-10

**Authors:** Gemma D. Traviss, Robert M. West, Allan O. House

**Affiliations:** Academic Unit of Psychiatry and Behavioural Sciences, Leeds Institute of Health Sciences, University of Leeds, Leeds, United Kingdom; University of Cape Town, South Africa

## Abstract

**Objective:**

To identify factors associated with infant growth up to 6 months, with a particular focus on maternal distress, and to explore the effect of ethnicity on any relation between maternal distress and infant growth.

**Methods:**

Cohort study recruiting White and Pakistani women in the United Kingdom (UK). Infant growth was measured at birth and 6 months. Standard assessment of mental health (GHQ-28) was undertaken in pregnancy (26–28 weeks gestation) and 6 months postpartum. Modelling included social deprivation, ethnicity, and other known influences on infant growth such as maternal smoking and alcohol consumption.

**Results:**

Maternal distress improved markedly from pregnancy to 6 months postpartum. At both times Pakistani women had more somatic and depression symptoms than White women. Depression in pregnancy (GHQ subscale D) was associated with lower infant growth at 6 months. Self-reported social dysfunction in pregnancy (GHQ subscale C) was associated with lower gestational age.. Pakistani women reported higher GHQ scores during pregnancy associated with smaller infants at birth. They lived in areas of higher social deprivation, reported less alcohol consumption and smoking postnatally, all independent influences on growth at 6 months.

**Conclusions:**

Maternal mental health in pregnancy is an independent influence on infant growth up to 6 months and is associated with ethnicity which was itself associated with deprivation in our sample. There is a complex relationship between symptoms of maternal distress, ethnicity, deprivation, health behaviours, and early infant growth. Measures should include both emotional and somatic symptoms and interventions to reduce risks of poor early growth need to include psychological and social components.

## Introduction

We are interested in the prevalence and characteristics of mental health problems in pregnancy and especially in any differences associated with ethnicity. One reason for this interest is the reported relationship between maternal mood disorder and pregnancy outcomes. Here we report our findings in relation to one such outcome – infant growth up to 6 months of age.

About 10–15% of women in western societies experience depression or anxiety around childbirth [1], [2] with up to 40% scoring above cut-off on common self-report measures of distress.[3] Recent findings suggest prevalences for diagnosable depression may be as high as 25% for some groups such as South Asian mothers [4], [5]. One relatively neglected question is the frequency and nature of somatic symptoms in pregnancy and their relation to other symptoms of distress. The most widely used standardised psychological measure used in pregnancy is the Edinburgh Postnatal Depression Scale [6], which omits somatic symptoms due to their similarity with the physiological symptoms associated with childbearing. There is however evidence to suggest an association between emotional and somatic symptoms in pregnancy [7], [8] and for increased somatic presentations of distress in South Asian women [9].

Undiagnosed and untreated distress during pregnancy has been shown to have risks for the mother and has been associated with poor nutrition (particularly in low-income countries) [10], [11] inadequate prenatal self-care, substance use, and intentional harm to oneself or the foetus [12] Symptoms of depression and anxiety have been linked with impaired neonatal development, including; developmental delay, behavioural disturbances [13] and cognitive impairment [14]–[16] and have been shown to be a predictor of mood disorders in mother postnatally [17]–[19] and in the child [20].

Research into maternal mood disorder and infant growth has indicated a link between depression and pre-term delivery, low birth weight [21] and reduced child growth [22] - probably the best single indicator of the infant's general health [23]. The association is stronger for women from developing countries and those of lower socioeconomic status [21], both of whom are at higher risk of experiencing major or minor antenatal depression [4], [5], [24], [25]. Studies comparing Indian women living in India and the United Kingdom (UK) suggest that socio-economic circumstances and environmental stresses such as infection and poor nutrition contribute to birth outcome. Babies born to more affluent mothers in India are of comparable weight to those in the UK [26]. The relation between antenatal distress, environmental and social factors, ethnicity and infant growth in deprived areas of developed countries such as the UK, remains unclear.

The relationship between maternal mental health and child growth may not simply be one between maternal depression and reduced growth. The findings are far from clear. Disturbances in feeding behaviour of depressed mothers are thought to affect both tails of the growth distribution, causing either stunting or obesity [27], with suggested differences attributed to the affluence of the country [28], [29].

There have been suggestions that early life environments are influential in the aetiology of obesity, and that infant obesity persists into adulthood [30], so it is important to understand the early risk factors for obesity and develop effective prevention interventions for pregnant women and their offspring. One candidate as a risk for infant obesity is maternal depression. A recent systematic review and meta-analysis have shown a bi-directional relationship between depression and obesity in adults [31]. Although the causal mechanisms are far from clear, the association raises questions about symptoms of depression around pregnancy and their effect on both maternal and infant size. We are interested in this relationship – and wanted to study it in South Asian as well as white women, not least because children from South Asian origin are at increased risk of obesity due to greater central adiposity and insulin resistance [30].

There is little relevant research in multi-ethnic populations living in developed countries such as the UK. Our study was conducted in West Yorkshire, where the ethnic diversity and level of deprivation allowed us to explore some of the potential factors associated with both maternal mental health and child growth up to 6 months, comparing women of White and Pakistani origin. Our aims were specifically to: 1) determine the prevalence of distress experienced around the time of pregnancy in this population, 2) to identify the influence of maternal distress on infant growth up to 6 months, after adjustment for the main candidate confounders, and 3) explore the association between maternal distress and infant growth in two ethnic groups, to determine whether distress (and especially somatic distress) is more common in South Asian women and if it is associated with lower infant growth in South Asian women in the UK.

## Methods

### Ethics Statement

The study was approved by Bradford Research Ethics Committee (07/H1302/112) and Research Governance was obtained from Bradford Teaching Hospitals NHS Foundation Trust and Bradford and Airedale Teaching Primary Care Trust. Written informed consent was provided by all participants prior to study involvement. The manuscript was prepared in line with the STROBE guidelines (See STROBE Checklist S1)

### Setting

The city of Bradford, West Yorkshire, is amongst the most deprived cities in the UK. Indices of Multiple Deprivation (IMD), based on seven deprivation domains, rank Bradford thirty-second out of 354 districts in England, with an average IMD score of 32 (a higher score indicating increased deprivation). It has a population of approximately half a million residents, 25% of whom are of South Asian origin (mainly Pakistani, but include Indian, Bangladeshi and Sri Lankan) and almost half of babies born have South Asian parents. The district has a wide range of public health problems associated with socioeconomic deprivation including rates of infant mortality and morbidity that are almost double the national average [32]. Rates of obesity are higher than the national average, with healthy eating and physical activity among the lowest.

### Participants

The Born in Bradford (BiB) cohort study aimed to recruit 12,000 mothers and their babies in order to investigate the prevalence and determinants of a range of child and adult diseases, see www.borninbradford.nhs.uk . All women accessing antenatal services at Bradford Royal Infirmary between autumn 2006 and 2010, were approached about recruitment into the study, with 85% uptake, see Raynor [33] for further details. Reasons for non-participation included language barriers, having another appointment to attend, lack of time, child commitments and feeling unwell. The women reported here comprised a consecutive sub-sample of the Born in Bradford cohort (*n* = 1716) recruited between August 2008 and April 2009. The sample represented the main ethnic groups in the area. Pakistanis make up the majority of the South Asian population in Bradford; the current analysis focussed specifically on White women indigenous to the UK and Pakistani women, including both 1^st^ and 2^nd^ generation citizens. Women were excluded if there was no suitable interpreter or translated study measures or they planned to move out of the area before the due date.

### Procedure

Women were recruited from those booked to deliver at Bradford Royal Infirmary. Written informed consent was taken by a trained project worker and then measures were taken at four time points during the study - at the routine booking appointment with the midwife (8–12 weeks gestation), at baseline which was conducted as part of the glucose tolerance testing appointment (26–28 weeks gestation), at birth and follow-up at 6 months postpartum. Study materials were translated into Urdu or transliterated into Mirpuri and project workers were trilingual to assist all participants in completing assessments. While a high proportion of participants were Pakistani, most chose to answer questions in English.

At birth babies were weighed andmeasured. Mothers were contacted by post at approximately 6 months postpartum and invited to return with their babies to the Bradford Royal Infirmary or booked for a home visit by their health visitor, to complete follow-up measures.

### Measures

#### Booking (8–12 weeks gestation)

Mothers weight and height were measured by the midwife at the booking appointment.

#### Baseline (26–28 weeks gestation)

The BiB baseline questionnaire was administered with mothers verbally by a project worker in their chosen language at baseline (26–28 weeks gestation). This was a 42 page questionnaire which took approximately 45 minutes to complete. Items included demographic data; ethnicity, history and current residential situation, family, education, employment, financial, diet, lifestyle and also a standardised measure of distress the General Health Questionnaire - 28 (GHQ-28) [34].

The GHQ-28 [34] is a standardised measure of distress and has been used with both clinical and non-clinical populations and also during pregnancy [3], [35]. We chose it because it includes both emotional and somatic symptoms. The measure comprises 28 items that relate to four sub-scales; A - somatic distress, B - anxiety, C - social dysfunction and D – severe depression. Items were scored using the Likert method (0-1-2-3), sub-scale scores were summed to provide a total GHQ score with a possible range of 0–84. The cut-off of 23/24 has been used in previous studies of both pregnant and non-pregnant women, as the threshold for caseness [3], [36]. We adopted this threshold, not as a means of diagnosing cases, but rather to gauge the level of distress. Studies have shown that the GHQ-28 is a valid method of case identification compared with the Clinical Interview Schedule and Present State Examination [36]–[38]. This has specifically been tested in our previous work with pregnant women of white and South Asian origin See Meer *et al* [39]. English, Urdu and Mirpuri versions of the GHQ-28 were used. The Urdu version had already been developed and showed satisfactory comparability to the original English version [40]. A transliterated Mirpuri version was developed by the BiB team. Anthropometric measures were also taken on mothers at baseline and 6 month follow-up, which included; weight, height, arm circumference and triceps skinfold thickness.

#### Birth

Babies were measured by a trained project worker. Weight, head, arm and abdominal circumference, subscapular and triceps skinfold thickness were measured. The International Obesity Task Force classifies obesity using body mass index (BMI). Baby length was not measured at birth due to the known difficulties and unreliability in keeping newborns fully stretched out and still for measurements to be performed [41], [42] . BMI could not therefore be calculated at this time point. Since abdominal circumference was measured at both time points and has been identified as a good indicator of body fat and fat distribution and as a suitable measure of infant growth [43], we chose that as our main measure of infant growth.

#### 6 month follow-up

The BiB baseline questionnaire (including GHQ-28) was administered with mothers and the anthropometric measures outlined above were repeated with babies at 6 months.

### Analysis

Characteristics of the cohort including GHQ scores were summarised by ethnicity, providing counts for categorical variables and means with standard deviations for continuous variables. Univariable comparisons were undertaken to compare the three ethnic groups, using Pearson's chi-square tests (χ^2^) and one-way analysis of variance (ANOVA) as appropriate. The GHQ subscales and GHQ total scores were considered as continuous variables. Pearson's correlations were used to assess correlations between somatic (GHQ subscale A) and emotional symptoms (GHQ subscale B+D), in pregnancy and at 6 months, by ethnicity.

Regression models were fitted using the R statistical computing software [44]. Covariate selection was made by selecting those sets of covariates which maximise the adjusted R^2^. Nonlinearity was explored using higher-order terms but only linear terms were necessary. When considering abdominal circumference, an analysis of covariance approach was preferred, to provide greater flexibility. While literature suggests smoking and alcohol consumption vary by ethnicity and are associated with preterm delivery and low birth weight [45], [46], interaction terms for these variables were not included in the models due to the extremely modest sample size of South-Asians engaging in these behaviours. For a direct comparison of total GHQ scores in pregnancy (26–28 weeks gestation) and 6 months post-partum, a matched pairs *t*-test was reported, since an analysis of covariance revealed no other significant associations.

Assuming a significance level of 5% with up to 5 predictors in a regression model, a small effect (Cohen's f^2^ = 0.02) can be detected with at least 90% power when the sample size exceeds 827 [47]. Participant numbers (*n*) are reported for each regression. Analyses were conducted on complete cases only.

## Results

### Participant Demographics

A total of 1716 women were recruited in pregnancy and 1247 (73%) mothers and their babies were followed up approximately 6 months after birth (mean follow-up = 6.7 months). Loss to follow-up was due to difficulty contacting families following childbirth. Mothers were contacted by mail and phone twice. Forty-eight percent of the baseline sample were Pakistani, 38% White and 14% Other. Participants had a mean age of 27.4 years (sd = 5.7) and weight of 67.2 kgs (sd = 16.0). The majority of women were married (75%), but there was a significant difference between the proportion of married White (42%) and Pakistani (99%) participants. The mean IMD score for the sample was 43.5 (sd = 18.4, range 4.8–78.2 with the worst reported for England being 89.2) [48]. This was higher in Pakistani mothers (48.9) than in White mothers (36.1). For 40% of mothers this was their first baby. The mean gestational age was 39.6 weeks and did not differ between groups. Mean birth weight was 3210 g, and was significantly lower in Pakistani and Other ethnicity groups. For further characteristics of the cohort, see Table S1.

### Maternal Mental Health

**Figure 1:**
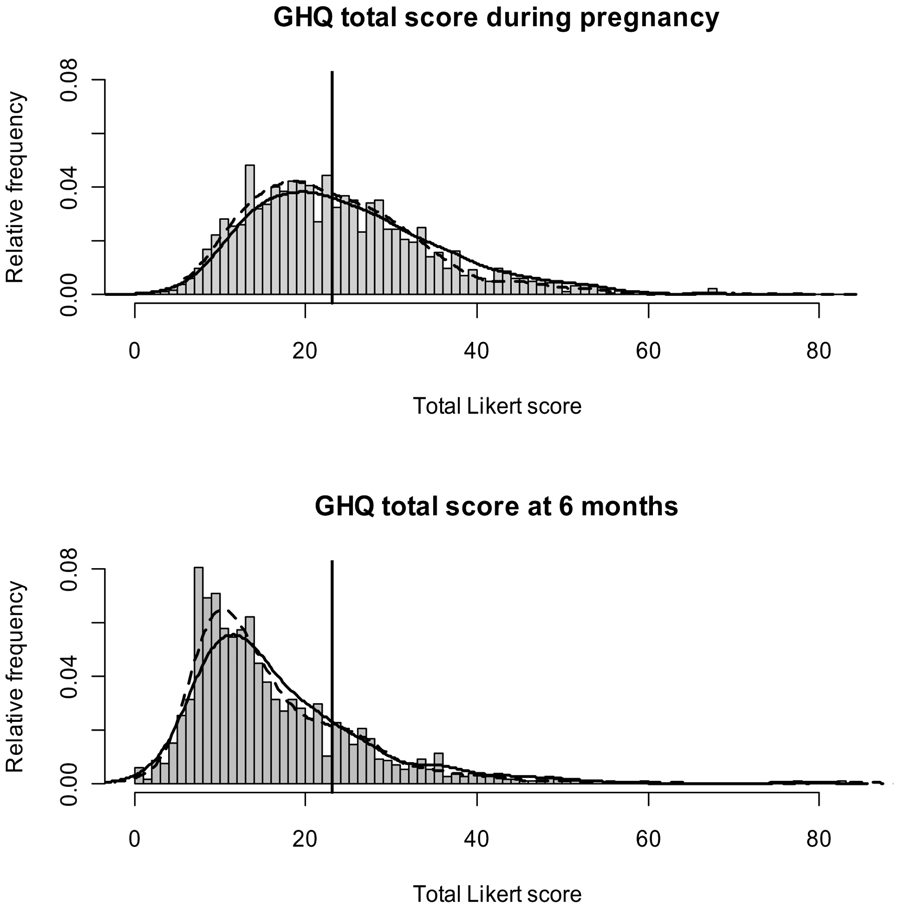
Histograms showing the distribution of GHQ total scores for the overall sample in pregnancy and 6 months. Estimators are for the overall sample, with the dashed line relating to White and the solid line, to Pakistani.

On GHQ total scores, the number of participants who exceeded cut-off ≥23 was 749/1602 (47%) in pregnancy and 229/1247 (19%) at 6 months postpartum. Overall, total GHQ scores were significantly reduced at 6 months (mean of diff = 8.0 (CI = 7.3, 8.6), df = 1124, p<0.001). Figure 1 shows the frequency distribution of GHQ total scores for the overall sample in pregnancy (26–28 weeks) and at 6 months, with suggested cut-off ≥23 and kernel smoothed estimates by ethnicity.

In relation to pregnancy GHQ total scores, regression analysis showed an association with marital status, ethnicity, and mothers BMI (n = 1080, adjusted R^2^ = 0.015) (Table 1). Being unmarried increased participants' GHQ score by around 3 points and being Pakistani, by around 4 points. The association with mothers BMI at booking was weak. An increase in BMI of almost 7 kg/m^2^ would be necessary for a 1 point increase on the GHQ. Pregnancy GHQ scores were the strongest predictor of 6 month GHQ scores (t(1124) = 23.7, p<0.001).

**Table 1:** Regression of GHQ Scores in Pregnancy.

*n* = 1080	Coefficient	95% CI	*Prob*
Intercept	21.2	(19.7, 22.8)	<0.001
Unmarried	2.85	(0.99, 4.71)	0.003
Ethnicity (Pakistani)	3.60	(1.84, 5.37)	<0.001
Ethnicity (Other)	2.44	(0.29, 4.59)	0.03
Mothers booking BMI	0.15	(0.04, 0.26)	0.01

Reference group White, married women.

Adjusted R^2^ = 0.015.

**Figure 2:**
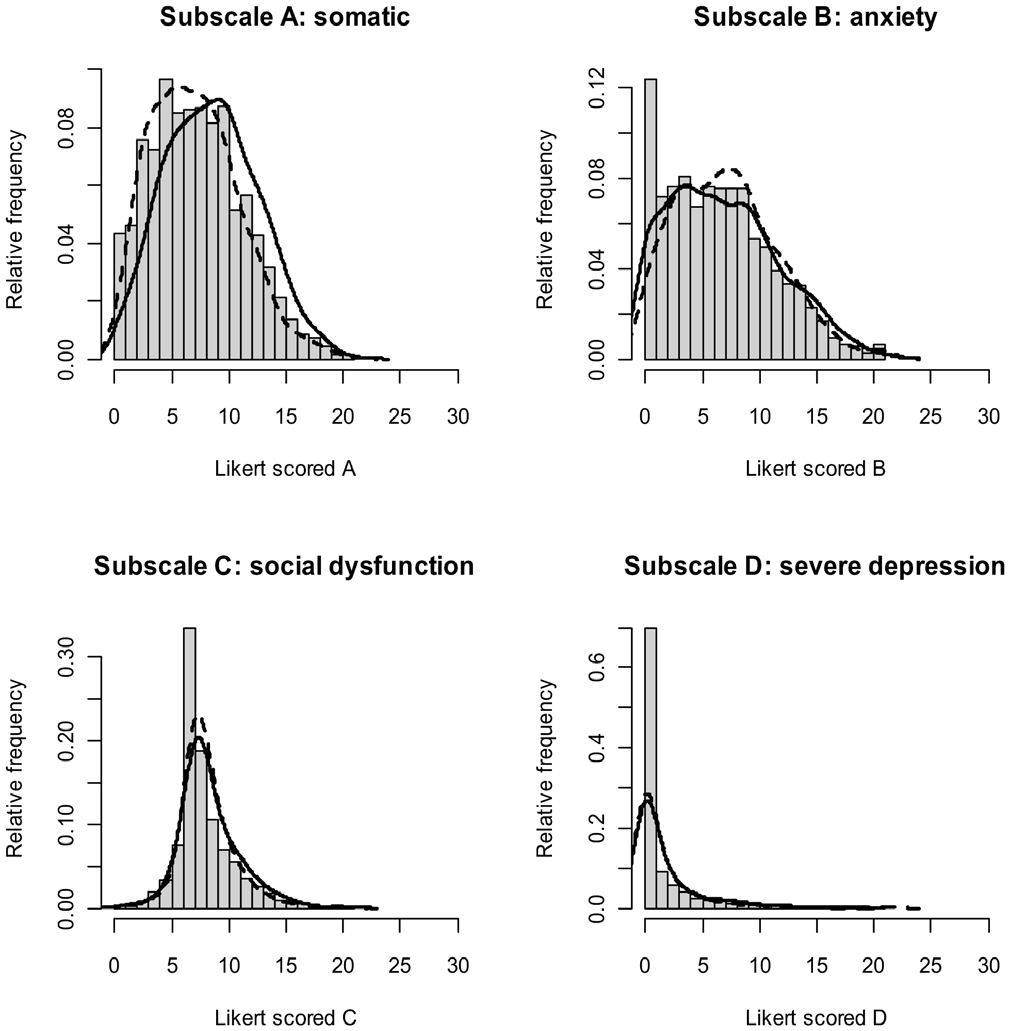
Kernel smoothed estimates of the probability densities of each GHQ subscale in pregnancy (26–28 weeks). Estimators are for the overall sample, with the dashed line relating to White and the solid line, to Pakistani.

**Figure 3:**
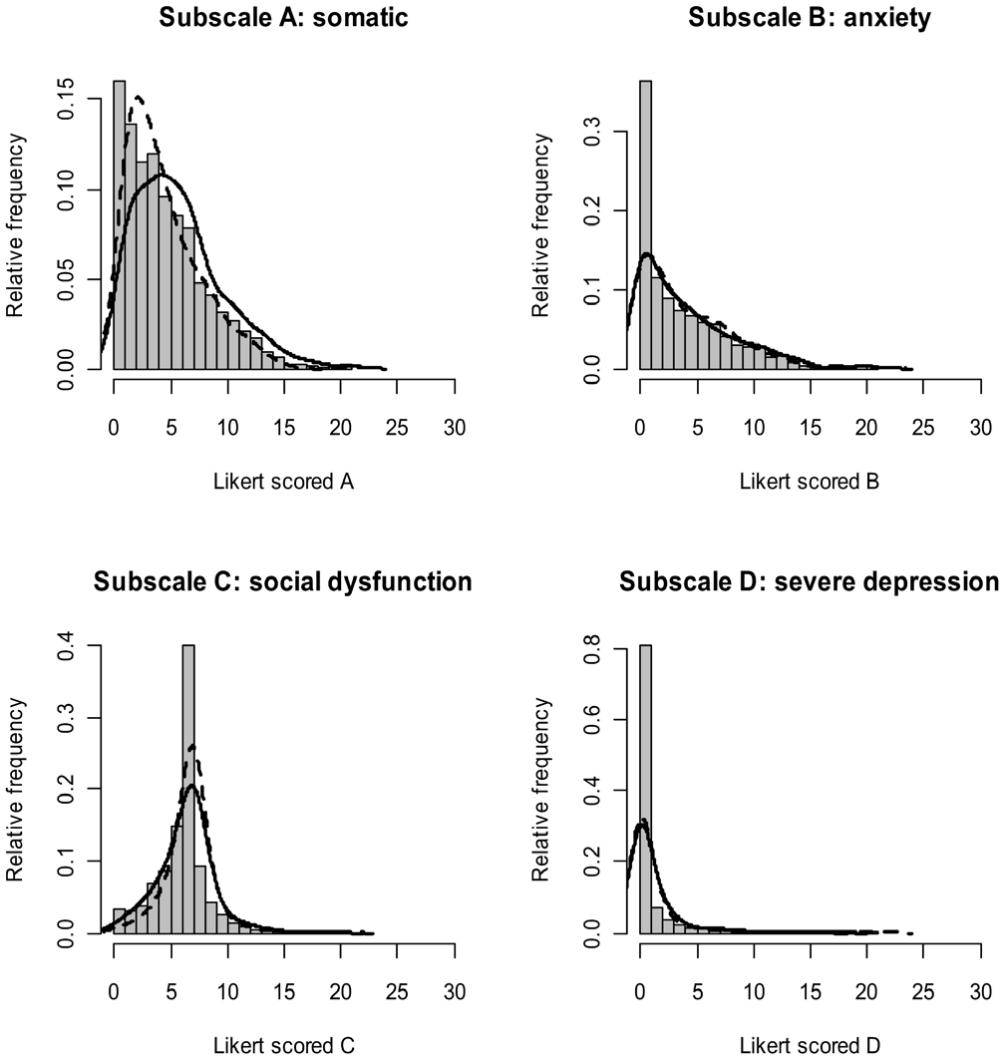
Kernel smoothed estimates of the probability densities of each GHQ subscale at 6 months postpartum. Estimators are for the overall sample, with the dashed line relating to White and the solid line, to Pakistani.

In pregnancy there was a significant difference between ethnic groups on GHQ subscales A (somatic) (*F*(2,1662) = 28.4, p<0.001) and D (depression) (*F*(2,1652) = 8.34, p<0.001), with Pakistani mothers scoring higher than White on both. Only subscale A remained significant at 6 months (*F*(2,1245) = 18.8, p<0.001). Pearson correlations for GHQ somatic and emotional symptoms (B+D) were r = 0.58 (0.52, 0.63) for White and r = 0.59 (0.54, 0.63) for Pakistani mothers in pregnancy, and r = 0.64 (0.58, 0.69) and r = 0.60 (0.54, 0.65) for White and Pakistani mothers at 6 months. Figures 2 and 3 show the frequency distribution of the four GHQ subscales for the sample in pregnancy and at 6 months postpartum with kernel smoothed estimates, by ethnicity. Subscale scores and test statistics for both time points are presented in Table S1.

### Infant Growth

#### Abdominal Circumference at 6 Months

Mean infant abdominal circumference at 6 months was 42.3 cm with a standard deviation of 3.0 cm (Table S1). The regression coefficient for birth abdominal circumference confirms that babies who were larger at birth are usually larger at 6 months. Consumption of alcohol since birth was associated an increase of 0.7 cm, not unsubstantial considering the standard deviation of 3.0 cm. Mother's BMI at 6 months was an influence; for each 10 kg/m^2^ greater BMI there was 0.3 cm increase in baby's abdominal circumference. Mother's self-reported smoking after pregnancy was associated with having a bigger baby at 6 months. Mother's GHQ subscale D score in pregnancy was negatively associated with 6 month infant abdominal circumference – each 10 points on the D subscale in pregnancy, was associated with a 1.0 cm lower abdominal circumference at 6 months. The model explains this variation with respect to the explanatory variables listed in Table 2, with the model chosen by the maximisation of adjusted R^2^ (0.074), the fit given accounting for R^2^ = 0.079 and being based on 1038 babies for whom complete records were available.

**Table 2:** Regression of Abdominal Circumference at 6 Months.

*n* = 1038	Coefficient	95% CI	*Prob*
Intercept	32.95	(30.10, 35.80)	
Abdominal circumference at birth	0.224	(0.151, 0.297)	<0.001
Mother consumed alcohol since birth	0.70	(0.28, 1.11)	0.001
Subscale D at visit per unit score	−0.104	(−0.179, −0.029)	0.007
Mother's BMI at follow-up	0.033	(0.003, 0.062)	0.029
Age of baby at follow-up	0.211	(−0.032, 0.453)	0.089
Mother regularly smokes	0.34	(−0.12, 0.80)	0.144

Adjusted R^2^ = 0.074.

#### Abdominal Circumference at Birth

Our model for 6 month abdominal circumference indicated that it was positively associated with postnatal BMI, postnatal drinking of alcohol and negatively associated with depression scores in pregnancy. Because abdominal circumference at birth was also associated with 6 month abdominal circumference, we sought associations with infant birth size to understand further the link between maternal mental health and infant growth. The mean abdominal circumference at birth was 31.2 cm with a standard deviation of 2.4 cm (Table S1). Babies born to Pakistani mothers were on average 0.63 cm smaller in abdominal circumference at birth than those born to White mothers. A higher deprivation of 40 IMD points was associated with babies having an abdomen 0.4 cm smaller, whereas each extra 20 kg on mother's weight at booking was associated with an increased infant abdominal circumference at birth of only 0.3 cm. Male babies were on average 0.35 cm larger than female babies. An increase of 0.48 cm in birth abdomen size was associated with each week of gestational age. The model explains this variation with respect to the variables listed in Table 3 and was chosen by the maximisation of adjusted R^2^ (0.138), the fit given, accounting for R^2^ = 0.142 and being based on 1333 babies for whom complete records were available.

**Table 3:** Regression of Abdominal Circumference at Birth.

*n* = 1333	Coefficient	95% CI	*Prob*
Intercept	11.53	(8.27, 14.79)	
Estimated gestational age per week	0.484	(0.403, 0.566)	<0.001
Pakistani	−0.63	(−0.91, −0.34)	<0.001
Other ethnicity	−0.44	(−0.82, −0.06)	
Mother's booking weight per kg	0.015	(0.007, 0.023)	<0.001
IMD (2007) for mother's residence	−0.009	(−0.016, −0.002)	0.009
Baby is male	0.35	(0.11, 0.59)	0.004

Adjusted R^2^ = 0.138.

#### Gestational Age

We explored further the link between maternal mental health and gestational age. Mean gestational age was 39.6 weeks with a standard deviation of 1.7 weeks (Table S1). An increase in mother's pregnancy GHQ subscale C score of 10 points was associated with a decrease in gestational age of 2.5 days. Similarly an increase in mother's age of 10 years was associated with a decrease in gestational age of almost 1 day. The model explains this variation with respect to the explanatory variables listed in Table 4. The model was chosen by the maximisation of adjusted R^2^ (0.004), the fit given accounting for R^2^ = 0.005 and being based on 1647 babies for whom complete records were available.

**Table 4:** Regression of Gestational Age.

*n* = 1647	Coefficient	95% CI	*Prob*
Intercept	40.2	(39.8, 40.7)	
Subscale C per unit score	−0.036	(−0.069, −0.003)	0.035
Mother's age per year	−0.013	(−0.028, 0.002)	0.082

Adjusted R^2^ = 0.004.

## Discussion

Our study was conducted in Bradford which is amongst the most deprived cities in the UK and has a high ethnic diversity. In it we aimed to explore the nature of maternal mental health problems in the two main ethnic groups in the city, and the association with infant growth up to six months.

Almost half (47%) of women in our study scored above cut-off on the GHQ in pregnancy. This is higher than the rate reported by Swallow [3], however their study participants were recruited at an earlier stage of pregnancy, 98% were Caucasian and information on deprivation was not reported. Our regression analysis showed an association of GHQ score with ethnicity. Pakistani and mothers of Other ethnicity scored up to four points higher on the GHQ, which is consistent with the literature that suggests higher prevalences for antenatal depression in South Asian women [4], [5]. In the sample overall GHQ scores decreased over the follow-up period, but pregnancy scores were a predictor of scores at 6 months, and ethnic differences in subscale A remained significant. Results showed a moderate correlation between somatic and emotional symptoms, in line the previous literature [7], [8]. The higher somatic scores in Pakistani women were associated with higher emotional distress levels; the relation between different GHQ symptoms did not vary by ethnicity in this sample. We thus found less convincing support for the notion of somatisation in South Asian women suggested by Wilson et al. [9] and others, but our results do support the importance of using an assessment tool that includes somatic symptoms to screen for psychological distress both during and following pregnancy.

We found that although GHQ score at 6 months was not associated with infant size at 6 months, GHQ subscale D (depression) scores in pregnancy were inversely related to infant size at 6 months. We found a relation between maternal distress and gestational age that may be mediated by social dysfunction during pregnancy. Social dysfunction related to feelings that things were taking longer to do, inability to make decisions and failing to play a useful part in things. Our results suggest that even in deprived areas of developed countries such as the UK, there is a relation between maternal depression and reduced infant growth [21], [22].

Pakistani women reported more symptoms on both subscales A (somatic) and D in pregnancy and also had significantly smaller babies at birth and 6 months. The difference may be even more pronounced, given the slightly later follow-up age of the Pakistani group. Although ethnicity was associated with both GHQ scores and birth size, there were other explanatory variables, which at the highest estimates only explained 14% of the variance. Previous studies showed a link between maternal mood and feeding behaviours [27], [49], [50], studying which was beyond the scope of this study. Therefore, our findings provide no evidence for a link between maternal mood and early excess infant weight gain [27].

These results were not explained entirely by confounding, since we adjusted for the main candidates. Thus, abdominal circumference at birth was a positive predictor of abdominal circumference at 6 months, and both ethnicity and deprivation scores were associated with smaller abdominal circumference at birth. Mothers from Pakistani and Other ethnic groups, delivering in the UK, had smaller babies compared to White women, as did those from more deprived areas. Deprivation was higher in the Pakistani and Other groups and therefore, as suggested by Margetts et al. [26] reduced birth size may be a reflection in part of factors related to deprivation and not solely to ethnicity itself. In our study however, ethnicity remained an independent risk after adjusting for deprivation. It is worth noting that the mean IMD score for the sample was substantially higher than that of Bradford generally (sample = 43.5, Bradford = 32), which suggests that mothers delivering at Bradford Royal Infirmary are from the most deprived areas of the city and that more affluent mothers perhaps choose to deliver elsewhere.

Self-reported postnatal maternal alcohol consumption and smoking were unexpectedly associated with larger babies at 6 months. Both behaviours were almost exclusively reported in the White cohort, which would be expected given the cultural and religious acceptability of both smoking and alcohol consumption in South Asian cultures. We do not know the amount or type of alcohol consumed since birth. Mother's booking BMI was also identified as a weak, but nonetheless positive predictor of maternal GHQ score which supports the work of Luppino et al. [31], suggesting a reciprocal link between obesity and depression.

The current study was not designed to look at mediators and further work is warranted in this area.

The current study has several notable strengths. It was a large prospective birth cohort study which looked at distress in both the antenatal and postnatal period, and its association with infant growth in a multi-ethnic sample. At 6 months, 73% of the sample were followed-up, which is in line with that of previous studies [28], [51], [52] (53–90%) and suggests little effect of ethnicity on uptake. We used a standardised assessment of psychological distress, available in the relevant languages and validated for use during pregnancy. Researchers working on the project were trilingual, which enabled the inclusion of wide range of participants, however, a minority of women who spoke only Bengali, Sylheti, Gujerati or Pushto were excluded.

There are several limitations to the study. The first is the use of abdominal circumference as a measure of infant growth. The time of assessment (pre or post feed) was not controlled for and therefore could vary somewhat in babies, however the use of abdominal circumference in babies has proved to be reasonably reliable [41]. BMI was not used due to the reported difficulty and unreliability in measuring baby length and height [42]. For adults, thresholds for healthy weight are lower for South East Asians, and the same may be true for babies. Although the sample was diverse in terms of ethnicity, one of the study's weaknesses was that the researchers did not have access to information regarding immigration status for all participants i.e. whether participants were first or second generation citizens. Although the literature is mixed, it suggests there may be a difference in birth weight of infants from first and second generation Asian mothers, with the most recent literature suggesting lower birth weight in second generation South Asian babies [26], [53]. Furthermore, while the rate of follow-up at 6 months was relatively high, it is still not known whether the failure to contact individuals could have been a result of increased levels of distress in non responders, that may have had a systematic influence on outcome. Regression analysis showed those lost to follow-up were the more deprived and younger mothers, with no association shown for ethnicity or any component of the GHQ at the 5% significance level. Finally, as mentioned earlier, only a small amount of variance in infant growth has been explained by the models (7.4% at 6 months), which suggests perhaps a more complex relationship exists between particular aspects of maternal distress, ethnicity, deprivation and infant growth and indicates a need to explore further contributory variables.

In conclusion, we have shown that maternal mental health is an independent influence on infant growth, with depressive symptoms in pregnancy (directly) and symptoms of social dysfunction (via an influence on gestational age) being associated with smaller infants at 6 months. Ethnicity had a complex relationship with factors influencing growth. Pakistani women had poorer mental health and more social deprivation while White women reported more smoking and drinking. Our findings suggest that when assessing mental health during and after pregnancy it is useful to record somatic symptoms as well as those of depression and anxiety, since they may be a good indicator of the psychological status of mothers. Further work is needed to explore potential mediating variables between maternal distress and infant growth, such as feeding behaviours and factors associated with deprivation in order to help identify mothers and babies at risk of compromised growth and to develop suitable interventions including both psychological and social components to support them.

## Supporting Information

Checklist S1 **STROBE Checklist.**
(DOC)

Table S1 **Characteristics of the Cohort.** * General Health Questionnaire (GHQ). Subscale A = Somatic symptoms, B = Anxiety, C = Social dysfunction, D = Depression. Scores on each subscale can range from 0–21 and for the GHQ total, from 0–84. ** IMD - Index of Multiple Deprivation.
(DOCX)
