# Supplementary material for: Maternal Mental Health and Its Association with Infant Growth at 6 Months in Ethnic Groups: Results from the Born-in-Bradford Birth Cohort Study
Source: PLoS One. 2012 Feb 10;7(2):e30707. doi: 10.1371/journal.pone.0030707 (PMC3277587; doi:10.1371/journal.pone.0030707)
Supplement: Table S1 — Characteristics of the Cohort. * General Health Questionnaire (GHQ). Subscale A = Somatic symptoms, B = Anxiety, C = Social dysfunction, D = Depression. Scores on each subscale can range from 0–21 and for the GHQ total, from 0–84. ** IMD - Index of Multiple Deprivation. (DOCX) [file pone.0030707.s002.docx]

| **Characteristic** | **White** | **Pakistani** | **Other** | **All ethnicities** | ***F*/χ^2^** | ***p-value*** |
| --- | --- | --- | --- | --- | --- | --- |
| Number recruited  Count (% cohort) | 648 (38%) | 810 (48%) | 244 (14%) | 1702  (14 unknown) |  |  |
| Number followed up at 6 months  Count (% of ethnic group) | 464 (72%) | 612 (76%) | 171 (70%) | 1247 (73%) | χ^2^=4.34 | 0.114 |
| Age of baby at Follow-up in months (sd) | 6.5 (0.7) | 6.7 (0.7) | 6.7 (0.7) | 6.7 (0.7) | *F*^2, 1283^=10.1 | <0.001 |
| Mother’s age  Mean (sd) | 26.63 (6.18) | 27.75 (5.13) | 28.27 (5.71) | 27.41 (5.67) | *F*^2, 1680^=10.5 | <0.001 |
| First baby | 315 (49%) | 254 (31%) | 110 (45%) | 686 (40%) | χ^2^=47.9 | <0.001 |
| Mother single – unmarried | 279/479 (58%) | 8/623 (1%) | 38/179 (21%) | 327/1294 (25%) | χ^2^=465.9 | <0.001 |
| Pregnancy GHQ-A* | 7.0 (3.8) | 8.5 (4.0) | 7.1 (4.4) | 7.7 (4.0) | *F*^2, 1662^=28.4 | <0.001 |
| Pregnancy GHQ-B | 7.1 (4.4) | 6.9 (4.8) | 6.9 (5.0) | 7.0 (4.7) | *F*^2, 1668^=0.52 | 0.592 |
| Pregnancy GHQ-C | 8.1 (2.3) | 8.3 (2.6) | 8.3 (2.9) | 8.2 (2.5) | *F*^2, 1665^=1.78 | 0.168 |
| Pregnancy GHQ-D | 1.3 (2.6) | 1.6 (2.8) | 2.2 (3.5) | 1.6 (2.9) | *F*^2, 1652^=8.34 | <0.001 |
| Pregnancy GHQ Total | 23.4 (10.2) | 25.0 (11.0) | 24.4 (12.5) | 24.3 (10.9) | *F*^2, 1592^=3.99 | 0.019 |
| Pregnancy GHQ above 23 | 271 (44%) | 368 (49%) | 105 (46%) | 749 (47%) | χ^2^=3.34 | 0.188 |
| 6-month GHQ- A | 4.3 (3.2) | 5.6 (3.8) | 4.6 (3.4) | 5.0 (3.6) | *F*^2, 1245^=18.8 | <0.001 |
| 6-month GHQ- B | 3.9 (4.0) | 4.0 (4.0) | 3.7 (3.8) | 3.9 (4.0) | *F*^2, 1224^=0.21 | 0.807 |
| 6-month GHQ- C | 6.7 (2.1) | 6.3 (2.4) | 6.2 (2.5) | 6.4 (2.3) | *F*^2, 1254^=4.61 | 0.010 |
| 6-month GHQ- D | 0.8 (2.2) | 1.0 (2.3) | 1.1 (2.2) | 1.0 (2.3) | *F*^2, 1242^=1.19 | 0.306 |
| 6-month GHQ Total | 15.8 (9.2) | 16.8 (9.8) | 15.6 (8.9) | 16.3 (9.4) | *F*^2, 1186^=1.86 | 0.157 |
| 6-month GHQ above 23 | 82 (18%) | 115 (20%) | 31 (19%) | 229 (19%) | χ^2^=0.86 | 0.652 |
| Gestational age in weeks (sd) | 39.6 (1.9) | 39.5 (1.6) | 39.7 (1.5) | 39.6 (1.7) | *F*^2, 1680^=0.54 | 0.585 |
| Birth weight in grams (sd) | 3322 (585) | 3127 (524) | 3188 (476) | 3210 (549) | *F*^2, 1680^=23.2 | <0.001 |
| Abdominal circumference at birth in cm (sd) | 31.8 (2.4) | 30.8 (2.4) | 31.1 (2.4) | 31.2 (2.4) | *F*^2, 1485^=27.5 | <0.001 |
| Abdominal circumference at follow-up in cm (sd) | 42.8 (3.0) | 42.0 (3.0) | 42.1 (3.1) | 42.3 (3.0) | *F*^2, 1244^=10.5 | <0.001 |
| Mother’s BMI at follow-up kg/m^2^ (sd) | 27.3 (6.4) | 26.4 (5.6) | 26.4 (5.5) | 26.8 (5.9) | *F*^2, 1247^=3.59 | 0.028 |
| Mother has consumed alcohol since birth | 385/479 (80%) | 4/631 (1%) | 46/178 (26%) | 437/1292 (34%) | χ^2^=779.9 | <0.001 |
| Mother is regular smoker during pregnancy | 236/479 (49%) | 34/631 (5%) | 35/179 (20%) | 307/1293 (24%) | χ^2^=292.2 | <0.001 |
| Mother’s weight at booking in kg (sd) | 72.3 (17.5) | 64.0 (14.2) | 64.9 (14.1) | 67.2 (16.0) | *F*^2, 1502^=49.4 | <0.001 |
| IMD** | 36.1 (19.0) | 48.9 (15.7) | 45.4 (18.7) | 43.5 (18.4) | *F*^2, 1699^=98.1 | <0.001 |
| Sex of baby is male | 313/641 (49%) | 389/802 (49%) | 125/240 (52%) | 827/1683 (49%) | χ^2^=0.99 | 0.611 |
